# Supplementary material for: Comparison of pre-treatment with different diluted sufentanil in reducing propofol injection pain in gastrointestinal endoscopy: A randomized controlled study
Source: PLoS One. 2025 May 29;20(5):e0325113. doi: 10.1371/journal.pone.0325113 (PMC12121801; doi:10.1371/journal.pone.0325113)
Supplement: S1 Table — (DOCX) [file pone.0325113.s001.docx]

**S1 Table. Definitions of adverse events during the painless gastrointestinal endoscopy**

| **Adverse Events** | **Definition** |
| --- | --- |
| Hypoxia | Oxygen saturation <90% and requiring sustained jaw thrust and/or increased oxygen flow[1]. |
| Cough | A sudden enhancement of expiratory airflow accompanied by typical cough sound during endoscopy examination, instead of drug-induced cough[2]. |
| Hypotension | Systolic blood pressure (SBP) < 90 mmHg, a mean arterial pressure < 65 mmHg, or an SBP decrease > 40 mmHg from baseline[3]. |
| Bradycardia | A heart rate less than 60 beats per minute during continuous heart monitoring[4]. |
| Dizziness | An unpleasant disturbance of spatial orientation or to the erroneous perception of movement[5]. |
| Nausea and vomiting | Patients experiencing nausea or vomiting within 24 hours after painless gastrointestinal endoscopy[6]. |

**References**

1. Leslie K, Allen ML, Hessian EC, Peyton PJ, Kasza J, Courtney A, et al. Safety of sedation for gastrointestinal endoscopy in a group of university-affiliated hospitals: a prospective cohort study. British Journal of Anaesthesia. 2017;118(1):90-9. https://doi.org/10.1093/bja/aew393

2. Plevkova J, Kollarik M, Poliacek I, Brozmanova M, Surdenikova L, Tatar M, et al. The role of trigeminal nasal TRPM8-expressing afferent neurons in the antitussive effects of menthol. J Appl Physiol (1985). 2013;115(2):268-74. https://doi.org/10.1152/japplphysiol.01144.2012

3. Ng PY, Sin WC, Ng AK, Chan WM. Speckle tracking echocardiography in patients with septic shock: A case control study (SPECKSS). Crit Care. 2016;20(1):145. https://doi.org/10.1186/s13054-016-1327-0

4. Hendriksen LC, Omes-Smit G, Koch BCP, Ikram MA, Stricker BH, Visser LE. Sex-based difference in the effect of metoprolol on heart rate and bradycardia in a population-based setting. J Pers Med. 2022;12(6):870. https://doi.org/10.3390/jpm12060870

5. Kadanka Z, Jr., Kadanka Z, Sr., Jura R, Bednarik J. Vertigo in patients with degenerative cervical myelopathy. J Clin Med. 2021;10(11):2496. https://doi.org/10.3390/jcm10112496

6. Kwon YS, Choi JW, Lee HS, Kim JH, Kim Y, Lee JJ. Effect of a Preoperative Proton Pump Inhibitor and Gastroesophageal Reflux Disease on Postoperative Nausea and Vomiting. J Clin Med. 2020;9(3). <https://doi.org/10.3390/jcm9030825>
