# Supplementary material for: Comparison of pre-treatment with different diluted sufentanil in reducing propofol injection pain in gastrointestinal endoscopy: A randomized controlled study
Source: PLoS One. 2025 May 29;20(5):e0325113. doi: 10.1371/journal.pone.0325113 (PMC12121801; doi:10.1371/journal.pone.0325113)
Supplement: S2 Table — (DOCX) [file pone.0325113.s002.docx]

**S2 Table. The Recovery Time Among Four Groups**

| Groups | 0µg/ml  group  (n=106) | 0.5µg/ml  group(n=104) | 1µg/ml group  (n=107) | 5µg/ml group  (n=104) | P  Value* |
| --- | --- | --- | --- | --- | --- |
| Recovery  Time(min) | 14.59±3.92 | 15.13±3.20 | 14.27±3.06 | 15.57±3.24 | **0.029** |

*：One way ANOVA analysis
