# Supplementary material for: Comparison of pre-treatment with different diluted sufentanil in reducing propofol injection pain in gastrointestinal endoscopy: A randomized controlled study
Source: PLoS One. 2025 May 29;20(5):e0325113. doi: 10.1371/journal.pone.0325113 (PMC12121801; doi:10.1371/journal.pone.0325113)
Supplement: S3 Table — (DOCX) [file pone.0325113.s003.docx]

**S3 Table. Pairwise Comparisons of Recovery Time Among Four Groups**

| Comparison | Standard Error | P Value* | 95%CI |
| --- | --- | --- | --- |
| 0µg/ml vs 0.5µg/ml | 0.465 | 0.254 | [-1.44, 0.38] |
| 0µg/ml vs 1µg/ml | 0.462 | 0.484 | [-0.58,1.23] |
| 0µg/ml vs 5µg/ml | 0.465 | **0.037** | [-1.89,-0.06] |
| 0.5µg/ml vs 1µg/ml | 0.464 | 0.066 | [-0.06,1.77] |
| 0.5µg/ml vs 5µg/ml | 0.467 | 0.344 | [-1.36,0.48] |
| 1µg/ml vs 5µg/ml | 0.464 | **0.005** | [-2.21,-0.38] |

*Post hoc pairwise comparisons were performed using Fisher’s least significant difference (LSD) test
