# Supplementary material for: Comparison of pre-treatment with different diluted sufentanil in reducing propofol injection pain in gastrointestinal endoscopy: A randomized controlled study
Source: PLoS One. 2025 May 29;20(5):e0325113. doi: 10.1371/journal.pone.0325113 (PMC12121801; doi:10.1371/journal.pone.0325113)
Supplement: S4 Table — (DOCX) [file pone.0325113.s004.docx]

**S4 Table. Total Propofol Consumption in Four Groups**

| Groups | 0µg/ml  group  (n=106) | 0.5µg/ml  group(n=104) | | 1µg/ml group  (n=107) | 5µg/ml group  (n=104) | P  Value* |
| --- | --- | --- | --- | --- | --- | --- |
| Total  Propofol  Consumption  (mg) | 218.5±36.8 | 196.7±31.0 | 183.8 ±25.0 | | 189.6 ±31.4 | **<0.001** |

*：One way ANOVA analysis
