# Supplementary material for: Comparison of pre-treatment with different diluted sufentanil in reducing propofol injection pain in gastrointestinal endoscopy: A randomized controlled study
Source: PLoS One. 2025 May 29;20(5):e0325113. doi: 10.1371/journal.pone.0325113 (PMC12121801; doi:10.1371/journal.pone.0325113)
Supplement: S5 Table — (DOCX) [file pone.0325113.s005.docx]

**S5 Table. Pairwise Comparisons of Total Propofol Consumption Across Four Groups**

| Comparison | Standard Error | P Value* | 95%CI |
| --- | --- | --- | --- |
| 0µg/ml vs 0.5µg/ml | 4.32 | **<0.001^a^** | [13.31, 30.31] |
| 0µg/ml vs 1µg/ml | 4.29 | **<0.001^a^** | [26.32, 43.19] |
| 0µg/ml vs 5µg/ml | 4.32 | **<0.001^a^** | [20.43, 37.43] |
| 0.5µg/ml vs 1µg/ml | 4.31 | **0.003** | [4.47, 21.42] |
| 0.5µg/ml vs 5µg/ml | 4.34 | 0.102 | [-1.42, 15.66] |
| 1µg/ml vs 5µg/ml | 4.31 | 0.178 | [-14.30,2.65] |

*Post hoc pairwise comparisons were performed using Fisher’s least significant difference (LSD) test
