# Supplementary material for: Comparison of pre-treatment with different diluted sufentanil in reducing propofol injection pain in gastrointestinal endoscopy: A randomized controlled study
Source: PLoS One. 2025 May 29;20(5):e0325113. doi: 10.1371/journal.pone.0325113 (PMC12121801; doi:10.1371/journal.pone.0325113)
Supplement: S6 File — (DOCX) [file pone.0325113.s006.docx]

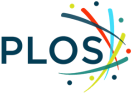


Lab Protocol Article

**Title** **Comparison of Pre-treatment with Different Diluted Sufentanil in Reducing Propofol Injection Pain**

Metadata

| Title | Comparison of Pre-treatment with Different Diluted Sufentanil in Reducing Propofol Injection Pain |
| --- | --- |
| Study Objective | This study aimed to investigate the effect of pre-treatment with different diluted sufentanil in reducing propofol injection pain in gastrointestinal endoscopy. |
| Participating Sites | Affiliated Hospital 2 of Nantong University |
| Study Design | A Randomized Controlled Study |
| Sample Size | 464 |
| Study Population | Inclusion criteria were as follows:Patients aged 18 to 65 years who underwent elective painless gastrointestina­l endoscopy was recruited.All of them were inserted with an intravenous infusion needle in the right dorsum prior to examination. |
|  | Exclusion criteria were as follows:  1.Patients with severe respiratory, cardiovascular, neurological, hepatic or renal disease (ASA physical status IV and V).  2.Patients who were allergic to propofol or sufentanil.  3.Patients with a preoperative assessment of difficult airway or BMI ≥30kg/m^2^.  4.Patients who were suffering chronic pain or long-term taking analgesic drugs.  5.Patients who were punctured larger antecubital vein.  6.Patients who needs to undergo endoscopic polyp treatment or a diagnostic ultrasound gastroscopy.  7.Patients who decline to participate.8.Patients with bradycardia. |
| Intervention | Patients were randomly divided into four groups： 0 µg/mLgroup(0.9% normal saline)，0.5 µg/mLgroup，1 µg/mLgroup and 5 µg/mLgroup.  Thirty seconds after the injection of sufentanil or normal saline, patients were injected with propofol. |
| Primary Endpoint | Numerical Rating Scale(NRS) . |
| Secondary Endpoint | Recovery time ,total propofol consumption and adverse events includin­g hypoxia, cough, hypotension, bradycardia, dizziness, nausea, and vomiting. |
| Statistical Analysis | Statistical analysis was executed using SPSS 24.0 and PASS 15.0.Measurement data that conforms to the gaussian distribution were presented as mean±SD, and categoric-al data were expressed by the number of cases or the percentage(%).Continuous data were analyzed using one-way analysis of variance.Categorical data were analyzed using the Chi-square tests or Fisher’s exact test,and Z test was used for further comparison between two groups.One-way ANOVA was used for comparison between multiple groups, and  LSD-t   test was used for further comparison between two groups.The numerical scoring  of pain(NRS) were compared among the four groups using Kruskal-Wallis Test,and all Pairwise test was used for further comparison between two groups.All statistical tests  are 2-tailed; the corrected differences were considered significant at P < 0.05. |
| Study Duration | From anesthesia clinic visit until hospital discharge |

**Funding**

Q.S was supported by the Health Commission of Nan-tong City Health and Family Planning Scientific Research Projects ( QNZ2023026) and The High-Level Scientific Research Project Cultivation Fund of Nantong First People's Hospital (YPYJJYB24008) .

L.Y was supported by the Health Commission of Nan-tong City Health and Family Planning Scientific Research Projects（MS2024033）and Nantong Municipal Science and Technology Scientific Research Projects(MSZ2024097).

**Competing interests**

All authors disclosed no relevant relationships.

**Data availability**

Data will be stored in the electronic database without indicating the name of the patients (a numeric code will be used).

**Associated content**

Qian Su. et al. (2025). Comparison of pre-treatment with different diluted sufentanil in reducing propofol injection pain in gastrointestinal endoscopy: a randomized controlled study.protocols.io.

[https://doi.org/10.17504/protocols.io.14egn4mwmv5d/v1](https://doi.org/10.17504/protocols.io.14egn4mwmv5d/v1).

**Abstract**

**Background:** This study aimed to investigate the efficacy of pre-treatment with different concentrations of sufentanil in mitigating propofol injection-induced pain.

**Methods:** In total, we randomly divided 421 patients who were scheduled for gastrointestinal endoscopy into four groups: 0 µg/mLgroup(0.9% normal saline)，0.5 µg/mLgroup，1 µg/mLgroup and 5 µg/mLgroup.

**Expected Results:**  Compared to the control group, other groups showed significantly reduced pain after propofol injection ,especially the 1 µg/mLgroup . Compared to the control group, 1 µg/mLgroup had significantly lower rates of cough, hypotension, and dizziness.

**Introduction**

A recent nationwide survey showed that the overall number of gastrointestinal endoscopic procedures in China is 14 million per year[1]. The most frequently used sedation drug was propofol, which provides a shorter time to sedation, a faster recovery profile, and improved patient satisfaction.1While propofol brings several strengths in sedation for gastrointestinal endoscopic procedures, it has been reported that the prevalence of propofol-induced pain on injection (POPI) is very high, ranges from 28% to 90% in the adult populatio­n[2].Many studies have been conducted to prevent POPI by utilizing medications such as opioids, ketamine, and lidocaine[3-5].However, no single intervention has been proven to completely eliminate POPI in all patients.Prior research has indicated that escalating the dosage of opioids can effectively mitigate POPI,but also lead to more complications[6-7].Chung DH (2011) revealed that administering sufentanil(0.3ug/kg) as a pretreatment to patients prior to propofol injection resulted in a significant reduction in the incidences of pain[8].However, H C Alves (2007)revealed that increasing sufentanil dosage ,when administered concurrently with propofol,could lead to higher risk of respiratory depression and death in mice[9].

In some cases, opioid receptor agonists  have such low efficacy that they cannot achieve the maximum response that a full agonist does, even when occupying all the receptors present in the tissue[10].Some endogenous substances interacting with other sites on the receptor could impact the process of agonists binding to the orthosteric site, such as sodium ions (Na+) and water molecules[11-12]. Many studies have suggested that Na+ has a two-sided modulatory effect  on the μ-opioid receptor.In most conditions, Na+ was used as a negative allosteric modulator(NAM)[13-14] .However, recent study revealed that Na+ can also function  as positive allosteric modulator (PAM)[15]. Besides, the inactived opioid receptors were vulnerable to interior  andexterior water molecules[16].These finding suggestthat sodium ions (Na+) and water molecules may impact the process of opioids binding to the opioid receptors on cellmembranes.Previous studies also suggested that the presence of citric acid in sufentanil can lead to pain by increasing the activity of Acid-Sensing Ion Channel 1(ASIC)[17-18].An increase in the extracellular concentration of hydrogen ions can lead to enhance activity of the ASIC, while conversely, a decrease in hydrogen ion concentration may result in inactivity of them[19].These finding suggest that changing the hydrogenion concentration of solution may impact the activate process of ASIC .However, to the author's knowledge, there has been no research on the effect of different dilutions of sufentanil in alleviating propofol injection-induced pain.

This study aimed to investigate the effect of pre-treatment with different diluted sufentanil in reducing propofol injection pain in gastrointestinal endoscopy.We hypothesize that altering the concentration of sufentanil in saline may impact the analgesia effect.

**Materials and Methods**

**Selection of Patients**

**Inclusion criteria**

— 18–65 years old,

— patients classified as ASA I-III,

— Body mass index (BMI) <30 kg/m^2^,

— Participants who underwent gastrointestinal endoscopy

**Exclusion criteria**

— Patients with severe respiratory, cardiovascular, neurological, hepatic or renal disease (ASA physical status IV and V).

— Patients who were allergic to propofol or sufentanil.

— Participants with anticipated difficult airway (Mallampati III/IV) or obesity (BMI ≥30 kg/m²).

— Patients who were suffering chronic pain or long-term taking analgesic drugs.

— Patients who were punctured larger antecubital vein.

— Patients who require endoscopic polyp treatment or diagnostic endoscopic ultrasound (EUS).

— Patients who decline to participate.

— Participants with bradycardia (resting heart rate <60 bpm) were excluded.

**Preliminary Experimental Results**

We recruited 80 patients and randomly allocated them in a 1:1:1:1 ratio to four groups according to a computer-generated randomization schedule. The pain relief rates (including no pain and mild pain) for the four groups were 70%, 85%, 90%, and 85%, respectively (as shown in Table 1).

**Table 1 Preliminary Experiment**

| Degree of Pain,n  (%) | Normal Saline Group  (n=20) | 0.5 µg/mL  SF Group  (n=20) | 1 µg/mL  SF Group  (n=20) | 5 µg/mL  SF Group  (n=20) |
| --- | --- | --- | --- | --- |
| None  (0 point)* | 8（40%） | 12(60%) | 14(70%) | 13(65%) |
| Mild pain  (1 point) | 6(30%) | 5(25%) | 4(20%) | 4(20%) |
| Moderate pain  (2 point) | 4(20%) | 2(10%) | 2(10%) | 2(10%) |
| Severe pain | 2(10%) | 1(5%) | 0(0%) | 1(5%) |
| (3 point) |  |  |  |  |
|  |  |  |  |  |
| Pain relief rate(NRS≤1) | 14(70%) | 17(85%) | 18(90%) | 17(85%) |

* The degree of pain was assessed through the Numerical Rating Scale (NRS) with a 3-point system: 0 for no pain, 1 for mild pain, 2 for moderate pain, and 3 for severe pain.

SF: Sufentanil

Pain relief rate: This metric indicates the proportion of patients achieving an NRS score of 0 or 1, indicating those who experience significant pain alleviation.

**Statistical Plan**

**Sample size**

The study was conducted using the Chi-Square Tests function in the PASS I5.0 software to determine the sample size.The degrees of freedom for the test were 9, with a Type I error rate of 0.05, a two-sided test, and a Type II error rate of 0.2.According to the results of preliminary experiment,the effect size (W value) was set at 0.1974, resulting in a required sample size of 402. Taking into account a dropout rate of 15%, a total of 464 participants were included, with 116 participants in each group.

**Statistical analysis**

Statistical analysis was executed using SPSS 24.0 and PASS 15.0.Measurement data that conforms to the gaussian distribution were presented as mean±SD, and categoric-al data were expressed by the number of cases or the percentage(%).Continuous data were analyzed using one-way analysis of variance.Categorical data were analyzed using the Chi-square tests or Fisher’s exact test,and Z test was used for further comparison between two groups.One-way ANOVA was used for comparison between multiple groups, and LSD-t test was used for further comparison between two groups.The numerical scoring of pain(NRS) were compared among the four groups using Kruskal-Wallis Test.All Pairwise test was used for further comparison between two groups.All statistical tests are 2-tailed; the corrected differences were considered significant at P < 0.05.

**Randomization and blinding**

The randomization schedule was generated by EpiCalc 2000 software. Patients were randomly allocated in a 1:1:1: 1 ratio to four groups  according to a computer-generated randomization schedule.Each patient was given a unique ID number and their data was collected for analysis after being de-identified.

In this study, the drug preparation process was blinded by "envelope" method.Based on the randomization sequence inside the envelopes, a nurse prepared three different concentrations of sufentanil(Yichang Humanwell Pharmaceutical Co., China) by diluting it with a 0.9% saline solution for use in various groups during the trial:0 µg/mL group(0.9% normal saline)，0.5 µg/mL group，1 µg/mL group and 5 µg/mL group.

The 1% propofol formulation (20 mL) used in this study was provided by Liaoning Haisco Pharmaceutical Co., Ltd. (China). Both study participants and investigators, including gastroenterologists and anesthesiologists, remained blinded to the study group allocation throughout the trial.

In case of an emergency (e.g., unexpected rapid deterioration in the patient’s clinical status), attending anesthesiologists or endoscopists could request unmasking of the treatment allocation, or adjust drug administration if necessary. To maintain the overall quality, legitimacy, and integrity of the clinical trial, unblinding of the test drug may occur only in critical circumstances when severe adverse events happen and considered to be related tosufentanil administration. In this circumstance, the PI. fully documents and explains the reasons for unblinding in a report to the Institutional Review Board (IRB).

**Study interventions**

On arrival at the examination room, a 07-model intravenous infusion needle (specification parameter 0.7mm×25mm)was inserted into a vein on the dorsum of the patient’s right hand.All patients will receive supplemental oxygen at a flow of 3 L/min through a nasal cannula.Monitoring included pulse oximetry, ECG (lead II), and noninvasive blood pressure.The intravenous medication process is completed by two individuals.After one anesthesiologist (who was blinded to group allocation) administered the pretreatment drug 30 seconds later, the other anesthesiologist subsequently administered intravenous 1% propofol ( initial dose of 2.5 mg/kg) to the patients at a rate of 0.5ml/sec.A supplement dose of propofol was carried out at a dose of 0.5 mg/kg[20] if patients involuntarily moved during the examination.The administration of pharmaceutical agents to patients with over weigh(defined as BMI≥ 25kg/m^2^ )was according to lean body weight(LBM)[21-22].The anesthesiologist was required to inquire the patients about the degree of pain every 5 seconds,using a Numerical Rating Scale (NRS) with a 3-point system: 0 for no pain, 1 for mild pain, 2 for moderate pain, and 3 for severe pain[23].In the case of desaturation or unplanned low blood pressure,physicians could elevate the man-dible or use ephedrine (6mg iv.) as rescue therapy.During the gastrointestinal endoscopy, any adverse events were recorded (e.g. hypoxia,cough,hypotension, bradycardia, dizziness nausea and vomiting).

Adverse events were defined in a procedures manual. These events were chosen because they required skilled intervention and/or had significant patient or health service implications:

1.Hypoxia:oxygen saturation <90% and requiring sustained jaw thrust and/or increased oxygen flow[24].

2.Cough was defined as a sudden enhancement of expiratory airflow accompanied by typical cough sound during endoscopy examination,instead of drug-induced cough[25].

3.Hypotension was defined as systolic blood pressure (SBP) < 90 mmHg, a mean arterial pressure < 65 mmHg, or an SBP decrease > 40 mmHg from baseline during the painless gastrointestinal endoscopy[26].

4.Bradycardia was defined as a heart rate less than 60 beats per minute during continuous heart monitoring[27].

5.Dizziness was defined as  an unpleasant disturbance of spatial orientation or to the erroneous perception of movement [28].

6.Nausea and vomiting were defined as patients experiencing nausea or vomiting within 24 hours after painless gastrointestinal endoscopy[29].

**Expected results**

| **Objective(s)** | **Endpoint(s)** |
| --- | --- |
| Primary Objective(s)  The primary objective of this study aims at evaluating the effects of pretreatment of multiple diluted sufentanil on the severity of POPI. | Endpoint(s) for primary objective(s)  After administered the pretreatment drugs and propofol,the anesthesiologist was required to inquire the patients about the degree of pain every 5 seconds,using a Numerical Rating Scale (NRS) with a 3-point system: 0 for no pain, 1 for mild pain, 2 for moderate pain, and 3 for severe pain. |
| **Secondary Objective(s)** | **Endpoint(s) for secondary objective(s)** |
| Secondary objectives are to compare the recovery time , total propofol consumption and adverse events among groups. | The recovery time is defined as:After the endoscopy, the researcher observe and recorde the time for patients from consciousness to self-opening eyes.  Total propofol consumption is defined as After the examination, the researcher records the total amount of propofol used.  The adverse events includes hypoxia,cough,hypotension, bradycardia, dizziness nausea and vomiting. |

**Ethics declarations**

The Ethics Committee of The Affiliated Hospital 2 of Nantong University will assess the protocol and the written and dated approval signed by the Ethics Committee chairman will

be obtained. The study will be registered at the Chinese Clinical Trial Registry

(www.chictr.org.cn). This study will be conducted in accordance with the principles laid

down by the World Medical Assembly and all applicable amendments (Helsinki, 1964) and

the ICH guidelines for Good Clinical Practice. This clinical trial will be conducted in

compliance with international laws and regulations, and laws and regulations of China, as

well as any applicable guidelines.

**Supporting information**

Due to privacy protections, the raw data contains sensitive information, including the patient's hospitalization number, name, surgery date, and other details. Interested authors can apply to our hospital's ethics committee to obtain the original data.The contact person is Dr. Hongqing Xu , who can be reached at xuhongqing000@126.com and +86 159 5131 2678.

**Acknowledgements**

No applicable

**Authors’ contributions**

Conception and design of the research: Lei Yao

Acquisition of data: Qian Su, Lu Li,Xin Sun，Xuefeng Yang

Analysis and interpretation of the data: Shu He,Boxiang Du，Xin Sun，Xuefeng Yang

Statistical analysis:Qiansu, Shu He，Xiangqing Wei

Obtaining financing: Qiansu,Lei Yao

Writing of the manuscript: Qian Su,Shu He.

Critical revision of the manuscript for intellectual content: Lei Yao, Boxiang Du

All authors read and approved the final draft.

**References**

1. Zhou, S., Zhu, Z., Dai, W., Qi, S., Tian, W., Zhang, Y., Zhang, X., Huang, L., Tian, J., Yu, W., & Su, D. (2021). National survey on sedation for gastrointestinal endoscopy in 2758 Chinese hospitals. British journal of anaesthesia, 127(1), 56–64. https://doi.org/10.1016/j.bja.2021.01.028

2. Desousa K. A. (2016). Pain on propofol injection: Causes and remedies. Indian journal of pharmacology, 48(6), 617–623. <https://doi.org/10.4103/0253-7613.194845>

3. Iyilikci, L., Balkan, B. K., Gökel, E., Günerli, A., & Ellidokuz, H. (2004). The effects of alfentanil or remifentanil pretreatment on propofol injection pain. Journal of clinical anesthesia, 16(7), 499–502. https://doi.org/10.1016/j.jclinane.2004.01.005

4. Chu, T., Zhou, S., Wan, Y., et al. (2024). Comparison of remimazolam and propofol combined with low dose esketamine for pediatric same-day painless bidirectional endoscopy: a randomized, controlled clinical trial. Frontiers in pharmacology, 15, 1298409.https://doi.org/10.3389/fphar.2024.1298409

5. Heuss, L. T., Hanhart, A., Dell-Kuster, S.,et al. (2011). Propofol sedation alone or in combination with pharyngeal lidocaine anesthesia for routine upper GI endoscopy: a randomized, double-blind, placebo-controlled, non-inferiority trial. Gastrointestinal endoscopy, 74(6), 1207–1214. https://doi.org/10.1016/j.gie.2011.07.072

6. VanNatta, M. E., & Rex, D. K. (2006). Propofol alone titrated to deep sedation versus propofol in combination with opioids and/or benzodiazepines and titrated to moderate sedation for colonoscopy. The American journal of gastroenterology, 101(10), 2209–2217. https://doi.org/10.1111/j.1572-0241.2006.00760.x

7. Jalota, L., Kalira, V., George, E., Shi, Y. Y., Hornuss, C., Radke, O., Pace, N. L., Apfel, C. C., & Perioperative Clinical Research Core (2011). Prevention of pain on injection of propofol: systematic review and meta-analysis. BMJ (Clinical research ed.), 342, d1110. <https://doi.org/10.1136/bmj.d1110>

8. Chung, D. H., Kim, N. S., Lee, M. K., & Jo, H. K. (2011). The effect and optimal dose of sufentanil in reducing injection pain of microemulsion propofol. Korean journal of anesthesiology, 60(2), 83–89. <https://doi.org/10.4097/kjae.2011.60.2.83>

9. Alves HC, Valentim AM, Olsson IA, Antunes LM. Intraperitoneal propofol and propofol fentanyl, sufentanil and remifentanil combinations for mouse anaesthesia. Lab Anim. 2007;41(3):329-336. doi:10.1258/002367707781282767

10. Livingston KE, Traynor JR. Allostery at opioid receptors: modulation with small molecule ligands. Br J Pharmacol. 2018;175(14):2846-2856. doi:10.1111/bph.13823

11. Shang Y, LeRouzic V, Schneider S, Bisignano P, Pasternak GW, Filizola M. Mechanistic insights into the allosteric modulation of opioid receptors by sodium ions. Biochemistry. 2014;53(31):5140-5149. doi:10.1021/bi5006915

12. Hu X, Wang Y, Hunkele A, Provasi D, Pasternak GW, Filizola M. Kinetic and thermodynamic insights into sodium ion translocation through the μ-opioid receptor from molecular dynamics and machine learning analysis. PLoS Comput Biol. 2019;15(1):e1006689. Published 2019 Jan 24. doi:10.1371/journal.pcbi.1006689

13. Shang Y, LeRouzic V, Schneider S, Bisignano P, Pasternak GW, Filizola M. Mechanistic insights into the allosteric modulation of opioid receptors by sodium ions. Biochemistry. 2014;53(31):5140-5149. doi:10.1021/bi5006915

14. Hu X, Provasi D, Ramsey S, Filizola M. Mechanism of μ-Opioid Receptor-Magnesium Interaction and Positive Allosteric Modulation. Biophys J. 2020;118(4):909-921. doi:10.1016/j.bpj.2019.10.007

15. Ye L, Neale C, Sljoka A, et al. Mechanistic insights into allosteric regulation of the A2A adenosine G protein-coupled receptor by physiological cations. Nat Commun. 2018;9(1):1372. Published 2018 Apr 10. doi:10.1038/s41467-018-03314-9

16. Venkatakrishnan AJ, Deupi X, Lebon G, Tate CG, Schertler GF, Babu MM. Molecular signatures of G-protein-coupled receptors. Nature. 2013;494(7436):185-194. doi:10.1038/nature11896

17. Yang YL, Lai TW. Citric Acid in Drug Formulations Causes Pain by Potentiating Acid-Sensing Ion Channel 1. J Neurosci. 2021;41(21):4596-4606. doi:10.1523/JNEUROSCI.2087-20.2021

18. Zaremba M, Ruiz-Velasco V. Opioid-Mediated Modulation of Acid-Sensing Ion Channel Currents in Adult Rat Sensory Neurons. Mol Pharmacol. 2019;95(5):519-527. doi:10.1124/mol.118.114918

19.Farrag M, Drobish JK, Puhl HL, et al. Endomorphins potentiate acid-sensing ion channel currents and enhance the lactic acid-mediated increase in arterial blood pressure: effects amplified in hindlimb ischaemia. J Physiol. 2017;595(23):7167-7183. doi:10.1113/JP275058

20. Li M, Ke W, Zhuang S. Effect of intravenous lidocaine on propofol consumption in elderly patients undergoing colonoscopy: a double-blinded, randomized, controlled trial. BMC Anesthesiol. 2022;22(1):61. Published 2022 Mar 4. doi:10.1186/s12871-022-01601-z

21. WHO Expert Consultation. Appropriate body-mass index for Asian populations and its implications for policy and intervention strategies [published correction appears in Lancet. 2004 Mar 13;363(9412):902]. Lancet. 2004;363(9403):157-163. doi:10.1016/S0140-6736(03)15268-3

22. Members of the Working Party, Nightingale CE, Margarson MP, et al. Peri-operative management of the obese surgical patient 2015: Association of Anaesthetists of Great Britain and Ireland Society for Obesity and Bariatric Anaesthesia. Anaesthesia. 2015;70(7):859-876. doi:10.1111/anae.13101

23. Kwak K, Kim J, Park S, et al. Reduction of pain on injection of propofol:combination of pretreatment of remifentanil and premixture of lidocaine with propofol. Eur J Anaesthesiol. 2007;24(9):746-750.doi:10.1017/S026502150600233X

24. Leslie K, Allen ML, Hessian EC, et al. Safety of sedation for gastrointestinal endoscopy in a group of university-affiliated hospitals: a prospective cohort study. Br J Anaesth. 2017;118(1):90-99. doi:10.1093/bja/aew393

25. Plevkova J, Kollarik M, Poliacek I, et al. The role of trigeminal nasal TRPM8-expressing afferent neurons in the antitussive effects of menthol. J Appl Physiol (1985). 2013;115(2):268-274. doi:10.1152/japplphysiol.01144.2012

26.Ng PY, Sin WC, Ng AK, Chan WM. Speckle tracking echocardiography in patients with septic shock: a case control study (SPECKSS). Crit Care. 2016;20(1):145. Published 2016 May 14. doi:10.1186/s13054-016-1327-0

27. Hendriksen LC, Omes-Smit G, Koch BCP, Ikram MA, Stricker BH, Visser LE. Sex-Based Difference in the Effect of Metoprolol on Heart Rate and Bradycardia in a Population-Based Setting. J Pers Med. 2022;12(6):870. Published 2022 May 25. doi:10.3390/jpm12060870

28. Kadanka Z Jr, Kadanka Z Sr, Jura R, Bednarik J. Vertigo in Patients with Degenerative Cervical Myelopathy. J Clin Med. 2021;10(11):2496. Published 2021 Jun 4. doi:10.3390/jcm10112496

29. Kwon YS, Choi JW, Lee HS, Kim JH, Kim Y, Lee JJ. Effect of a Preoperative Proton Pump Inhibitor and Gastroesophageal Reflux Disease on Postoperative Nausea and Vomiting. J Clin Med. 2020;9(3):825. Published 2020 Mar 18. doi:10.3390/jcm9030825
