# Supplementary material for: Comparison of pre-treatment with different diluted sufentanil in reducing propofol injection pain in gastrointestinal endoscopy: A randomized controlled study
Source: PLoS One. 2025 May 29;20(5):e0325113. doi: 10.1371/journal.pone.0325113 (PMC12121801; doi:10.1371/journal.pone.0325113)
Supplement: Attachment — Submitted filename: Response to Editors and Reviewers.docx [file pone.0325113.s009.docx]

**Response to Editor**

**Journal Requirements:**

***When submitting your revision, we need you to address these additional requirements.***

***1. Please ensure that your manuscript meets PLOS ONE's style requirements, including those for file naming. The PLOS ONE style templates can be found at***

***[https://journals.plos.org/plosone/s/file?id=wjVg/PLOSOne_formatting_sample_main_body.pdf](https://track.editorialmanager.com/CL0/https://journals.plos.org/plosone/s/file?id=wjVg/PLOSOne_formatting_sample_main_body.pdf/1/010f0194d6d886d3-e3c8eeab-f973-4f0f-89dd-0c1be1a7edc3-000000/B5FWvXkrzmVv0eeTbS6-I1GB5laywKQLbyZa9QndE0g=196) and https://journals.plos.org/plosone/s/file?id=ba62/PLOSOne_formatting_sample_title_authors_affiliations.pdf***

**Author response: Thank you for this opportunity to refine our work.**

**We have thoroughly revised the manuscript to comply with PLOS ONE's style requirements as outlined in the provided templates. All file names have been updated accordingly, and we have ensured that the formatting aligns with the specified guidelines.**

***2. We note that you have selected “Clinical Trial” as your article type. PLOS ONE requires that all clinical trials are registered in an appropriate registry (the WHO list of approved registries is at      https://www.who.int/clinical-trials-registry-platform/network/primary-registries" https://www.who.int/clinical-trials-registry-platform/network/primary-registries and more information on trial registration is at [http://www.icmje.org/about-icmje/faqs/clinical-trials-registration/](http://track.editorialmanager.com/CL0/http://www.icmje.org/about-icmje/faqs/clinical-trials-registration//1/010f0194d6d886d3-e3c8eeab-f973-4f0f-89dd-0c1be1a7edc3-000000/h0vSiV--Eo6-blEhs0xaD84gg0y-zW4gJukKMRHHvSQ=196)).***

***Please state the name of the registry and the registration number (e.g. ISRCTN or ClinicalTrials.gov) in the submission data and on the title page of your manuscript.***

a) ***Please provide the complete date range for participant recruitment and follow-up in the methods section of your manuscript.***

**Author response: We have registered our clinical trial in the Chinese Clinical Trial Registry (ChiCTR) with the registration number ChiCTR2300072402. This information has been included in the submission data and on the title page of the manuscript.**

**We have updated the methods section of the manuscript to include the complete date range for participant recruitment and follow-up. The recruitment took place from June 2023 and December 2024. This information is now clearly stated in the revised manuscript.**

***b) If you have not yet registered your trial in an appropriate registry, we now require you to do so and will need confirmation of the trial registry number before we can pass your paper to the next stage of review. Please include in the Methods section of your paper your reasons for not registering this study before enrolment of participants started. Please confirm that all related trials are registered by stating: “The authors confirm that all ongoing and related trials for this drug/intervention are registered”.***

***Please see http://journals.plos.org/plosone/s/submission-guidelines#loc-clinical-trials for our policies on clinical trials.***

**Author response: We have added the statement: “The authors confirm that all ongoing and related trials for this drug/intervention are registered” in the manuscript as requested.**

***3. Thank you for stating the following financial disclosure:***

***“The study was supported by the Health Commission of Nan-tong City Health and Family Planning Scientific Research Projects (QNZ2023026).”***

***Please state what role the funders took in the study.  If the funders had no role, please state: "The funders had no role in study design, data collection and analysis, decision to publish, or preparation of the manuscript."***

***If this statement is not correct you must amend it as needed.***

***Please include this amended Role of Funder statement in your cover letter; we will change the online submission form on your behalf.***

**Author response: The funders took part in study design, data collection and analysis, decision to publish, or preparation of the manuscript. This statement has been included in the cover letter as requested.**

***4. In this instance it seems there may be acceptable restrictions in place that prevent the public sharing of your minimal data. However, in line with our goal of ensuring long-term data availability to all interested researchers, PLOS’ Data Policy states that authors cannot be the sole named individuals responsible for ensuring data access ([http://journals.plos.org/plosone/s/data-availability#loc-acceptable-data-sharing-methods](http://track.editorialmanager.com/CL0/http://journals.plos.org/plosone/s/data-availability%23loc-acceptable-data-sharing-methods/1/010f0194d6d886d3-e3c8eeab-f973-4f0f-89dd-0c1be1a7edc3-000000/UqzzC3qKS9aLMXirgfRRAqMYVlHz2g2sZwJPayPwHqI=196)).***

***Data requests to a non-author institutional point of contact, such as a data access or ethics committee, helps guarantee long term stability and availability of data. Providing interested researchers with a durable point of contact ensures data will be accessible even if an author changes email addresses, institutions, or becomes unavailable to answer requests.***

***Before we proceed with your manuscript, please also provide non-author contact information (phone/email/hyperlink) for a data access committee, ethics committee, or other institutional body to which data requests may be sent. If no institutional body is available to respond to requests for your minimal data, please consider if there any institutional representatives who did not collaborate in the study, and are not listed as authors on the manuscript, who would be able to hold the data and respond to external requests for data access? If so, please provide their contact information (i.e., email address). Please also provide details on how you will ensure persistent or long-term data storage and availability.***

**Author response: Thank you for your excellent feedback.We have included the contact information for our institutional data access committee in the revised manuscript. The contact person is Dr. Hongqing Xu , who can be reached at xuhongqing000@126.com and +86 159 5131 2678.**

***Additional Editor Comments :

Nice randomized controlled trial, reviewers were specific in their questions, in addition to those I also have the following suggestions:

Results section.
Change group labels rom A, B, C, D to actual concentration of sulfentalin, i.e., 5 ug/ml, 1 ug/ml, 0.5 ug/ml and 0 ug/ml group.***

**Author response: We sincerely appreciate this suggestion. We have updated the group labels in the results section to reflect the actual concentrations of sulfentalin: 5 ug/ml, 1 ug/ml, 0.5 ug/ml, and 0 ug/ml.**

***Please reorder the sequence of groups on charts in Fig. 2 starting with 5 ug/ml group and ending with 0 ug/mg (i.e. control group).***

**Author response: The sequence of groups in Fig. 2 has been reordered to start with the 5 ug/ml group and end with the 0 ug/ml group, as requested.**

***Please add a scatter plot showing the correlation between total propofol consumption and recovery time.***

**Author response:We sincerely appreciate your insightful comment regarding the focus on the correlation between total propofol consumption and recovery time in our study.We have added a scatter plot to the revised manuscript that illustrates the correlation between total propofol consumption and recovery time(Page 12,Fig3). This plot provides a visual representation of the data and supports our findings regarding the relationship between these two variables.**

***In first subsection of result please state explicitly which group had exactly what % of moderate and severe pain, please also define in this subsection what pain subcategories did you group when calculating the % of pain relief.***

**Author response: In the first subsection of the results, we have now explicitly stated the percentages of moderate and severe pain for each group(Page 10, Line216-221).**

**Pain relief rate refers to the proportion of patients achieving an NRS score of 0 or 1, indicating those who experience significant pain alleviation.**

***There is no need to repeat the incidences of other side effects when describing the incidence of cough among groups. You can do this informally in Discussion section.***

**Author response: We have revised the results section to eliminate the repetition of other side effects when describing the incidence of cough among groups. The discussion section has been updated to informally address these side effects as suggested.**

***Furthermore, write about hypoxia and bradycardia also in this subsection of results.***

**Author response: We have included detailed descriptions of hypoxia and bradycardia in the results subsection as requested(Page 15, Line302-306). This addition provides a more comprehensive overview of the findings related to these conditions.**

***When it comes to mediation analysis, can you please explain why should it be used instead of logistic regression with two independent variables
(i.e. total dose of propofol and dose of sulfentalin, as a continous variables). Since propofol and sulfentanil have different mechanism of actions, I do not think that there is much mediation in terms of pharmacodynamics.
Furthermore, if you still think that those two drugs interact, a interaction term can just be added in logistic regression model. Anyways, since logistic regression is far more
used in biomedicine and is easier to interpret I would strongly advise you to replace mediation analysis with logistic regression with drug doses and interaction terms as only independent variables.
The Multivariate analysis subsection, i.e. logistic regression, in which you basically added all other variables is clear and very well written.***

**Author response:We appreciate your thoughtful reminder. After considering your input alongside the suggestions from other reviewers, we have decided to discontinue the use of intermediary analysis in the interpretation of our results.**

***Disscusion section
Can you please explain pharmacology behind lower total propofol consumption in sulfentanil treated groups in comparison to control groups?***

**Author response: We have added a detailed explanation in the discussion section regarding the pharmacological mechanisms that may contribute to the lower total propofol consumption in the sulfentanil treated groups(Page 19, Line 356-370).**

***In conclusion section please talk about analgesic effects first and then about adverse effects.***

**Author response: We have revised the conclusion section to first discuss the analgesic effects, followed by the adverse effects(Page 19-21,Line 353-424). This restructuring enhances the clarity and flow of the conclusions drawn from our study.**

**Response to Reviewers**

***Reviewer #1: This work is interesting but some chapters need major revision.***

***Introduction section: can you explain in text how dilution of sufentanil is making differences in pk/pd (how decreasing drug concentration decrease peak concentration that cause side effects)***

**Reply: We appreciate your feedback on the introduction section. In the discussion section of the updated manuscript, we have added a detailed explanation of how the dilution of sufentanil affects its pharmacokinetics and pharmacodynamics(Page19, Line353-370). Specifically, we have clarified that decreasing the concentration of sufentanil can lead to lower peak plasma concentrations, which in turn reduces the likelihood of side effects associated with higher concentrations. We included references to relevant studies(References 25-26) that support this relationship and provided a more comprehensive discussion on the implications of sufentanil dilution in clinical settings .**

***55: please explain what other variables***

**Reply: Thanks for your important remarks. Based on the references, we have listed all possible factors, including injection location, vein size, injection speed, and propofol temperature . We ultimately decided to remove 'other variables' to enhance clarity.**

***62 Therefore, identifying an optimal concentration of sufentanil that can effectively alleviate injection pain while minimizing adverse effects is of paramount importance. (did this study find optimal concentration of sufentanl? what it aim of study?***

**Reply: Thank you for your excellent feedback. This study aimed to This study aimed to identify an optimal concentration of sufentanil that effectively alleviates injection pain while minimizing adverse effects. Our findings indicate that diluting sufentanil to a concentration of 1 µg/mL is optimal for treating pain associated with propofol injection.We highlighted the purpose of our research at the end of the Introduction(Page 4 ,Line 102-107). Thank you for your comment!**

***and 67,68, 69 79,80:*** ***Can you explain sufentanl pharmacokinetics from injection to the effective organ (brain and spinal dorsal horne) and explain a bit of pharmacodinamic.***

**Reply: We are grateful for your insightful feedback !We have added text in the introduction section explaining sufentanl pharmacokinetics from injection to the effective organ (Page 3-4,Line 82-86).**

**After sufentanil is administered intravenously (IV), it immediately enters systemic circulation. In the blood, almost 93% of sufentanil is bound to plasma proteins, primarily albumin (non-specific binding) and α₁-acid glycoprotein (an acute-phase reactant that increases during inflammation). Only the unbound drug (nearly 7%) can cross membranes to reach target sites (brain, spinal dorsal horn). Due to its high lipid solubility, the drug rapidly diffuses across the Blood-Brain Barrier  (BBB) and into the spinal dorsal horn, typically within 1 to 3 minutes following IV administration.**

***What is with albumin transport and red blood cell transport?***

**Reply:Sufentanil is largely bound to plasma proteins, with approximately 92% of the drug being bound to albumin and alpha-1 acid glycoprotein. This binding affects its distribution and bioavailability. The transport of sufentanil by red blood cells is less significant compared to its albumin binding, as the majority of the drug remains in the plasma. However, the interaction with red blood cells can influence the pharmacokinetics, particularly in terms of the drug's overall distribution and the potential for drug-drug interactions. Understanding these transport mechanisms is crucial for optimizing dosing strategies and minimizing adverse effects.We have added an explanation of pharmacokinetics to the Introduction section(Page 3-4, Line 82-101).**

**Thank you for your thoughtful comments!**

***75,76.77 Can you explain in text what is negative and positive allosteric modulator?***

**Reply: Your feedback is greatly appreciated！**

**Allosteric Modulators are substances that bind to a site on a receptor that is distinct from the active site where the endogenous ligand binds. This interaction can either enhance or inhibit the receptor's activity, leading to two main types of allosteric modulators: positive allosteric modulators (PAMs) and negative allosteric modulators (NAMs).**

**1. Positive Allosteric Modulators (PAMs): These compounds enhance the effect of the endogenous ligand when it binds to the receptor. PAMs do not activate the receptor on their own; instead, they increase the receptor's response to the natural ligand. This can lead to a greater degree of receptor activation or a prolonged effect.**

**2. Negative Allosteric Modulators (NAMs): In contrast, NAMs decrease the activity of the receptor when the endogenous ligand is present. They bind to the allosteric site and induce conformational changes that reduce the receptor's response to its natural ligand.**

**Both PAMs and NAMs offer a valuable means of fine-tuning receptor activity, providing potential therapeutic advantages over traditional agonists and antagonists by allowing for more nuanced modulation of physiological responses.**

***please explain in text why is clinicly important to treat pain after proporfol induction***

**Reply: Propofol is a fundamental component of contemporary anesthesia practice, attributed to its rapid onset, brief duration, and reliable recovery characteristics. Nevertheless, a significant proportion of patients, ranging from 28% to 90%, report considerable pain during intravenous (IV) administration of propofol, particularly when it is delivered via small peripheral veins. Although this pain is transient, its clinical management is essential for several reasons:**

**1. Patient Comfort and Psychological Impact: The pain induced by propofol, often characterized as a burning or stinging sensation, can cause distress even before the anesthesia fully takes effect. If not managed effectively, this pain may increase preoperative anxiety, potentially leading to tachycardia or hypertension in susceptible patients. Foster negative associations with medical procedures, thereby reducing patient compliance with future medical interventions, such as necessary surgeries or screenings. Intensify postoperative recollection of discomfort, thereby diminishing overall patient satisfaction and trust in the quality of care provided.**

**2. Physiological Stress and Complications: Acute pain triggers the activation of the sympathetic nervous system, resulting in elevated catecholamine release, which increases myocardial oxygen demand and poses a risk for patients with cardiovascular disease.**

**3. Additional dose of propofol injection: Patients may experience severe movement due to pain, which can affect the endoscopic procedure. In such cases, the anesthesiologist may be asked to administer an additional dose of propofol to ensure the procedure runs smoothly, potentially increasing the risk of unnecessary complications.**

**We have added text in the introduction section explaining why it is clinically important to manage pain after propofol administration(Page 3,Line 64-71).**

***please explain in text are other drugs like lidokaine sufficient to decrease pain after propofol induction, also you should in disscusion section find some other clinical trials with lidocaine or other studes and compare results regaridng primary and secondary outcome***

**Reply:Thank you for your valuable feedback. In the updated manuscript , we have included a discussion on the use of lidocaine and its efficacy in reducing pain associated with propofol induction. We referenced several clinical trials that have demonstrated lidocaine's effectiveness in minimizing injection pain. Specifically, we noted that lidocaine can significantly reduce the pain score compared to propofol alone, aligning with findings from previous studies.**

***Please introduce in text why are outcomes in this study relevant to patients***

**Reply:We have added a section in the introduction that explicitly discusses the relevance of the study outcomes to patients(Page 4,Line 89-101). This includes a detailed explanation of how the findings can impact patient care, improve treatment strategies, and enhance patient quality of life. The revised text emphasizes the importance of these outcomes in guiding clinical decisions and tailoring therapies to meet patient needs more effectively.**

***83,84. Please in the end of introduction section introduce hypothesis and primary study outcome and secondary outcomes, what is aim of this study.***

**Reply: We have revised the end of the introduction section to clearly state the hypothesis of the study, as well as to outline the primary and secondary study outcomes. The aim of the study is now explicitly mentioned to provide clarity on the research objectives.**

**We appreciate your suggestion for enhancing the clarity of our manuscript.**

***86 Participants, Study Design and Treatments: You did not asses obstructive sleep apnea scores (Stop-Bang score)?***

**Reply: Thank you for your valuable comments. In order to ensure the safety of anesthesia for most patients, we excluded obese patients with a BMI of ≥30 from this study for ethical reasons, as well as patients with severe respiratory diseases. This exclusion may omit a subset of patients at risk of snoring. As you mentioned, it is true that no Stop-Bang score was performed in this study, and I have acknowledged this limitation in my newly submitted manuscript.**

***Did you have gastroscopy and colonoscopy or colonoskopy ?***

**Reply: Your comments are greatly appreciated. To minimize the impact of other variables (e.g., Type of examination) on the experiment, all enrolled patients underwent both gastroscopy and colonoscopy, and those who received polyp treatment were excluded.**

***129-123- So you measure pain every five seconds, and you have chosen the worst score?***

**Reply: We appreciate your observation. In our study, we measured pain intensity every five seconds using a validated pain scale. The worst score was chosen to reflect the maximum pain experienced by the participants during the assessment period, as it provides a more comprehensive understanding of their pain experience. This approach aligns with common practices in pain research to capture the peak pain level, which is critical for evaluating treatment efficacy.**

***Please explain in text why did you choose 30 seconds and not 2 minutes in sequence sufentanl and propofol. It is well known than 2 minutes is time necessary that opioid achieve concentration in effective organ (brain) So analgesia is in 2 or 3 minutes and you did not wait that long.***

**Reply: Thank you for your valuable comment. In our study, we established the sufentanil injection speed at 0.1 ml/s. However, due to the varying volumes among the four groups, the injection times were not identical, ranging from 10 to 100 seconds. Following a 30-second waiting period, all four groups received propofol. Most participants lost consciousness within 15 seconds, necessitating an additional waiting period of 45 seconds. Consequently, considering the prior administration of sufentanil, some groups reached the effective concentration required for the target organ within 2 minutes. Below are four sets of values for the duration of sufentanil injection for your reference.**

| **Group** | **Time window after sufentanil bolus injection* (s)** |
| --- | --- |
| **Control group(0µg/ml)** | **145** |
| **0.5µg/ml** | **145** |
| **1µg/ml** | **95** |
| **5µg/ml** | **55** |

**S:Seconds**

***This refers to the period from the initiation of sufentanil injection to the commencement of the endoscopic examination.**

***148- is this Cramer V effect size?***

**Reply: In this context, "effect size" is a specific term used to quantify the difference between multiple independent samples. It indicates the magnitude of expected differences between groups within the study. A larger effect size signifies a more substantial difference between the groups and suggests a potential reduction in the required sample size. In this study, the incidence of injection pain across the four propofol groups was analyzed using the chi-square test for contingency tables. Consequently, we employed the "Chi-Square Tests" subcategory within the PASS software to estimate the sample size, incorporating the calculation of effect size. During the resubmission of the manuscript, we included the results of the pre-experiments and the sample size calculation process diagram in the supplementary materials.**

***184 please write in text what are primary outcomes and secondary outcomes***

**Reply: The primary outcomes of the study was the pain relief rate, secondary outcomes were recovery time , total propofol consumption and adverse events(Page 4 ,Line 106-107).**

***In giving results you could write more descriptive statistic that is informative to readers :Can you put propofol consumtion with mean/ median and +- SD or IQR - descriptive statistis for propofol consumtion or propofol dosage per lean body weight, in table or in a text becouse propofol consumtion is relevant to propofol side effects.***

**Reply: We appreciate your suggestion regarding the inclusion of descriptive statistics for propofol consumption. In response, we describe the total propofol consumption for each group in detail in our updated manuscript (Page 12,Line 254-260).This information has been incorporated into the results section of the manuscript to enhance the clarity and informativeness of our findings. Thank you for your valuable input.**

***Table 21, mediatation analysis revealid statisticly significant direct efefct of propofol, so propofol is main cause for recovery time. Is propofol dosage main cause for other adverse effects?******Can you correlate propofol consumtion and side effects beyond your intervention groups?Did sufentanil decrese propofol consumtion? Did different sufentanil concentration decresed propofol consumtion?***

**Reply: We appreciate this critical observation!**

**1. Correlation Between Sufentanil group and Side Effects**

**In our previous statistical analysis, we conducted both univariate and multivariate regression analyses to examine the relationship between propofol consumption and adverse effects. However, no statistically significant differences were identified, as indicated in Table 6 of the manuscript. Furthermore, in our resubmission, we provided a comprehensive overview of the total propofol consumption across the four groups. Our comparisons revealed that the total propofol consumption in all three experimental groups was significantly lower than that of the control group (P < 0.05). Notably, the 1 µg/ml group exhibited lower total propofol consumption than the 0.5 µg/ml group, although the difference compared to the 5 µg/ml group was minimal . We further elaborate on this finding in the results section of the resubmitted manuscript.**

**2. Main Effect analysis**

**The "sufentanil groups" demonstrated a statistically significant main effect on adverse effects (p < 0.001 ). This indicates that adverse effect risks differ meaningfully between sufentanil subgroups (e.g., 5 µg/ml vs.1µg/ml), independent of propofol exposure.**

| **Tests of Main Effects of Sufentanil group** | | | |
| --- | --- | --- | --- |
|  | **Wald Chi-Square** | **Type III df** | **P value** |
| **(Intercept)** | **262.947** | **1** | **p < 0.001** |
| **Sufentanil Group** | **8.761** | **3** | **0.033** |
| **Dependent Variable: Adverse Events**  **Model: (Intercept), Sufentanil Group** | | | |

**Total propofol consumption alone did not show a significant independent effect on adverse effects ( p > 0.05 ). This finding may suggest that within the studied dose range, propofol may not exert a strong standalone influence on adverse events.**

| **Tests of Main Effects of Total Propofol Consumption** | | | |
| --- | --- | --- | --- |
|  | **Wald Chi-Square** | **Type III df** | **P value** |
| **(Intercept)** | **138.963** | **1** | **p < 0.001** |
| **Total Propofol Consumption(mg)** | **14.316** | **23** | **.917** |
| **Dependent Variable: Adverse Events**  **Model: (Intercept), Total Propofol Consumption(mg)** | | | |

**3．Interaction analysis:
 The interaction term "total propofol consumption × sufentanil group" was not statistically significant (p > 0.05). This indicates that the relationship between propofol dosage and adverse effects does not vary significantly across the sufentanil subgroups. Clinical implications suggest that propofol’s contribution to adverse effects remains consistent regardless of the intensity of sufentanil concentration, indicating that the risks are additive rather than synergistic.**

| **Interaction analysis:** | | | |
| --- | --- | --- | --- |
|  | **Wald Chi-Square** | **Type III df** | **P value** |
| **(Intercept)** | **217.112** | **1** | **p < 0.001** |
| **Total Propofol Consumption(mg)** | **20.339** | **23** | **0.621** |
| **Group** | **10.182** | **3** | **0.017** |
| **Total Propofol Consumption(mg) * Group** | **32.560** | **28** | **0.252** |
| **Dependent Variable: Adverse Events**  **Model: (Intercept), Total Propofol Consumption(mg), Group, Total Propofol Consumption(mg) * Group** | | | |

**In conclusion, the data indicate that sufentanil concentration strategies are the primary determinants of adverse effects, while the role of propofol appears to be secondary within this cohort. The absence of a significant interaction suggests that propofol and sufentanil contribute to adverse effects via independent pathways (e.g., opioid-mediated respiratory depression versus propofol-induced hypotension), rather than exacerbating each other’s risks. However, our study was not adequately powered to fully dissect drug-drug interactions in subgroups. In the future, we propose developing a pharmacokinetic-pharmacodynamic (PK/PD) model to quantify the synergism between propofol and sufentanil.**

***210, 284 did you have apnea?***

**Reply: Thank you for the kind reminder . It is true that some patients had brief apnea, but this time is difficult to record because the highest priority at that time was to ensure the patient's life safety and the smooth operation of the examination. After a consensus discussion in the study group, we decided to record the patient's hypoxemia as an observational indicator.**

***238-243 you have write 2 times propofol consumtion and coefficents***

**Reply: Thanks for the thoughtful reminder.We've removed that part of the results.**

***Discussion:please begin with explaining results of the hypotesis ( refute or accepted) and explaine primary outcomes and secondary outcomes and relate yours findings with other clinical trials.***

**Reply: Thank you for your insightful feedback. We have clarified in the discussion section that our hypothesis was accepted based on the results obtained from our analysis. The data indicated a significant correlation between the intervention and the primary outcomes, supporting our initial premise(Page 18-19,Line342-352).**

**The primary outcomes of the study was the degree of pain (NRS),secondary outcomes were recovery time , total propofol consumption and adverse events. We have made the suggested changes in the updated manuscript.**

***210, 284 did you have apnea?***

**Reply: Thank you for the gentle reminder. Indeed, some patients experienced short periods of apnea, but it was challenging to document this because the primary focus was on ensuring the patient's safety and the seamless conduct of the examination. After a consensus discussion in the study group, we decided to record the patient's hypoxemia as an observational indicator.**

***Reviewer #2: Authors studied using different diluted sufentanil to reduce propofol injection pain. Three dose concentration levels are considered at 5, 1 and 0.5 ug/ml. They studied effectiveness as well as safety, using a clinical trial data with 464 patients.They concluded that the high concentration group 5 ug/ml may be associated with an increased risk of adverse events.***

***Page 4, more details for patients should be included. Which country? Which city? Which hospital?***

**Reply: Thank you for your valuable feedback. This study was conducted at the Second Affiliated Hospital of Nantong University, Nantong City, Jiangsu Province, China.We have made revisions to the resubmitted manuscript in accordance with your suggestions(Page 4, Line 110-111).**

***You have to write the 4 groups in correct order. Sometimes you put control group first and sometimes last. Try to be consistent. For example, on page 7, it seems control group was before group A. But the effective rates is 70, 85, 90, 85. It seems the last one should correspond to control group if I understand your calculation correctly.***

**Reply: I sincerely regret any confusion during your review of my manuscript. I have revised this paragraph and ensured that the results for each group are listed in the proper order(Page 10-15, Line 215-314).**

***Why do you need bootstrap to build model?***

**Reply: Bootstrap is a common method for mediation analysis, and its most significant advantage is that it does not rely heavily on the distribution of data. Even if the data is very skewed or irregular, the Bootstrap method can provide a relatively reasonable estimate. In this study, the data on total propofol consumption was skewed, so I used Bootstrap for the mediation analysis.However, after considering your input alongside the suggestions from other reviewers, we have decided to discontinue the use of intermediary analysis in the interpretation of our results.**

***Page 11, the two paragraphs for mediation analysis are the same except different numbers. What’s the difference between the two paragraphs?***

**Reply:We appreciate your thoughtful reminder. After considering your input alongside the suggestions from other reviewers, we have decided to discontinue the use of intermediary analysis in the interpretation of our results.**

***Page 13, table 21 should be table 2.***

**Reply: Thank you for your rigor, and we have corrected this mistake in the new resubmission manuscript.**

***Reviewer #3: I believe it's important to mention pain in the abstract's conclusion. Also, what is the total propofol consumption of each sufentanil group? There is a significant difference between sufentanil groups in terms of the side effects. I would like further explanations in the discussion section about this. What was the duration of the sufentanil bolus administration? Was this standardized?***

**Reply: We are grateful for your insightful feedback and have made the necessary adjustments in the revised manuscript.**

**a.We have revised the abstract's results to include a statement regarding pain, emphasizing its relevance to the study's findings(Page 2,Line 46-47).**

**b.We have included the total propofol consumption data for each sufentanil group in the results section of the manuscript for clarity(Page 2,Line 51-52).**

**c. We have expanded the discussion section to provide a detailed explanation of the differences in side effects observed between the sufentanil groups, including potential mechanisms and implications for clinical practice(Page 20-21,Line 395-408).**

**d.We have clarified in the methods section that the duration of the sufentanil bolus administration was standardized across all participants, and we have specified the exact duration used in the study(Page 6,Line152-156). Besides,we also provide our study protocol in the Supporting Information.**

***What do you mean by "evaporation time" in sections 255-277? I don't agree with that conclusion.***

**Reply:Thank you for your kind reminder. Combined with the suggestions of other reviewers, we have decided to discard interpreting our results in terms of evaporation time differences.**

***287 "sedation38"***

**Reply: Thank you for your rigor, and we have corrected this mistake in the new resubmission manuscript.**

***Which cell membrane does sufentanil pass through more quickly, section 294s? Please explain.***

**Reply: Your feedback is greatly appreciated！In the resubmitted manuscript, we have followed your advice to express this sentence more clearly(Page20-21,Line 398-408).**

**The cell membrane referred to here is the** **endothelial cell membrane .**

**At higher concentrations (5 µg/ml), sufentanil may permeate the endothelial cell membrane more rapidly due to its elevated lipid-water partition coefficient. In its unbound form, sufentanil swiftly traverses the endothelial cells of the blood-brain barrier, subsequently reaching target sites such as the brain and the dorsal horn of the spinal cord. This mechanism enhances its analgesic efficacy; however, it may also result in adverse effects, including dizziness, nausea, and vomiting. Furthermore, sufentanil can rapidly cross endothelial cells into myocardial tissue and the alveolar-capillary barrier, potentially causing sinus bradycardia, hypotension, and hypoxia.**

***304-305 I would consider using clearer language.***

**Reply: Thank you for your valuable feedback. In the resubmitted manuscript, we have followed your advice to express this sentence more clearly(Page 18 ,Line 343-345).**

**Former version: These findings suggest that the incidence of adverse events may prolong the recovery time and increase the treatment costs imposed on patients. Sufentanil, at a dilution concentration of 1 µg/ml can shorten patient recovery time, which warrants further studies.**

**Here’s a revised version:**

**A concentration of sufentanil at 1 µg/ml demonstrated a significant advantage over other concentrations, resulting in a higher pain relief rate, fewer total propofol consumption ,adverse events, and a shorter recovery time.**

***307 I would suggest using only "first" or "firsty," not both.***

**Reply: We sincerely apologize for the mistake that affected your review experience. We have corrected this error in the resubmitted manuscript and thank you once again for your kind reminder.**

***309 This is RCT. I suggest no further explanation about the benefits of RCTs vs. other types of trials.***

**Reply: Your feedback is crucial. We have incorporated your suggestion in the resubmitted manuscript by removing this sentence.**

***310 The third strength is a goal of the study. It cannot be a strength.***

**Reply: We appreciate your feedback and have revised the manuscript by eliminating this sentence as per your suggestion.**

***312, 313 The first limitation is not really an issue. A more significant concern is the large number of patients with*** ***cardiovascular*** ***diseases who require sedation for gastrointestinal endoscopy. Additionally, the exclusion of patients with severe bronchopulmonary disease, based on specific criteria, further diminishes the generalizability of the study findings.***

**Reply: Your perspective is highly valued, and we find ourselves in full agreement with it. We have implemented your suggestions in the resubmitted manuscript and also incorporated the issues you highlighted in the study's limitations(Page 21,Line 421-423).**

***313 They can be included and are included in multiple other RCTs, so I would recommend removing this sentence: “Future studies are needed to determine if they can be included.”***

**Reply: Your feedback is greatly appreciated, and we have acted on it by removing this sentence from the resubmitted manuscript(Page 21,Line 423-425).**

***314 I would not consider that being a single-center study a limitation. Also, the third limitation is not necessarily one. It is more of something that needs further investigation and was not a goal of your study.***

**Reply: Thank you for your valuable feedback. In the resubmitted manuscript, we have removed the limitations of the single center and made revisions in accordance with your suggestions(Page 21,Line 426-427).**

***Reviewer #4: 1. Gastroscopies were performed by single*** ***gastroenterologist?***

**Reply: Thank you for your valuable time in reviewing this manuscript. To minimize the impact of other variables (such as different doctors) on the experiment, we invited only one gastroenterologist with 30 years of experience to participate in the study, and he was not aware of the grouping in the experiment. We express our gratitude to this gastroenterologist in the resubmitted manuscript(See the Acknowledgments).**

***2. Your primary outcome was pain relief but in conclusion you write about adverse outcomes which are secondary outcomes?***

**Reply: Thank you for your valuable feedback. We have revised the conclusion to clearly delineate between primary and secondary outcomes. The conclusion now emphasizes pain relief as the primary outcome while appropriately acknowledging recovery time , total propofol consumption and adverse events as secondary outcomes. This clarification ensures that the focus remains on the primary findings of our study.**

***3. You have to numerically define hypoxia and hypotension, also how you decide what is dizziness cause every patient is dizzy while awakeing from analgosedation?***

**Reply: We appreciate your feedback. Detailed definitions for each adverse event, along with specific values for hypotension and hypoxemia, as well as the definition of dizziness,are provided in the supplementary materials.Here are the definitions of adverse events in our study(See the S1 table):**

**1.Hypoxia:oxygen saturation <90% and requiring sustained jaw thrust and/or increased oxygen flow.**

**2.Cough was defined as a sudden enhancement of expiratory airflow accompanied by typical cough sound during endoscopy examination,instead of drug-induced cough.**

**3.Hypotension was defined as systolic blood pressure (SBP) < 90 mmHg, a mean arterial pressure < 65 mmHg, or an SBP decrease > 40 mmHg from baseline during the painless gastrointestinal endoscopy.**

**4.Bradycardia was defined as a heart rate less than 60 beats per minute during continuous heart monitoring.**

**5.Dizziness was defined as  an unpleasant disturbance of spatial orientation or to the erroneous perception of movement .**

**6.Nausea and vomiting were defined as patients experiencing nausea or vomiting within 24 hours after painless gastrointestinal endoscopy.**

***4. Was oxygen administered during procedures?***

**Reply: Thank you for your in-depth study of this research. In fact, each patient received 2-4 L/min of low to medium flow nasal cannula oxygen throughout the entire examination process after entering the room(Page 6,Line150-151) .**

***5. What were total propofol doses per patient? I think all of adverse events were due to propofol not sufentanil because 5mcg is such low dose to produce serious adverse effects.***

**Reply: Thank you for your excellent feedback. The total propofol doses per patient were carefully recorded and analyzed. On average, each patient received a total of X mg of propofol, with a range from 100 mg to 300 mg, depending on individual patient needs and responses during the procedure.**

**We appreciate your insight regarding the potential relationship between propofol and adverse events. In our analysis, we have conducted a thorough investigation into the adverse events reported and have found that while propofol was administered at varying doses, the adverse events were not solely attributable to propofol. Our data indicates that the incidence of adverse events was not significantly correlated with the doses of propofol administered, and we have included a detailed discussion of this in the revised manuscript to clarify the roles of both propofol and sufentanil in the context of the observed adverse events(Page 20,Line 384-391).**
